# Editorial: a year of growth and opportunity

**DOI:** 10.29045/14784726.2026.3.10.4.1

**Published:** 2026-03-01

**Authors:** Julia Williams, Caitlin Wilson, Graham McClelland

**Affiliations:** Editor-in-Chief, *British Paramedic Journal*; Associate Editor, *British Paramedic Journal*; Associate Editor, *British Paramedic Journal*

This year marks the 25th anniversary of the College of Paramedics, 10 years since the first edition of the *British Paramedic Journal* (BPJ) and, quite significantly, the year in which the College evolves into the Royal College of Paramedics. These are not simply dates on a timeline; they represent milestones in a journey of collective action, perseverance and transformation across our profession.

When the College (formerly known as the British Paramedic Association) was founded in 2001, paramedicine in the UK was still emerging as an academic and clinical discipline. Research activity was limited or, dare we say, non-existent, career pathways were narrow and much of our practice relied on tradition rather than evidence. Since then, paramedicine has moved on and rapidly evolved. One marker of this evolution was the creation of the BPJ a decade ago by the College of Paramedics, which signalled a commitment to generating and disseminating evidence focusing on pre-hospital, urgent and emergency unscheduled care.

The growth of paramedic research in the UK is exciting. We have a rapidly expanding community of research paramedics, paramedic doctoral students, post-doctoral paramedic fellows, (associate) professors of paramedic science/paramedicine and research leads embedded in ambulance services. There is an increasingly sophisticated research infrastructure supporting paramedic research across the UK. While it is not there yet (whatever ‘there’ means), we have come a long way since we first started including research as a pillar of practice in our profession’s career pathways. Paramedics are now leading multi-centre trials, shaping (inter)national policy, partnering in interdisciplinary research collaborations and contributing to the global evidence base at a level that would have been a mere dream only a couple of decades ago.

This growth is not accidental. It reflects the maturity of a profession that has continually adapted to meet the needs of patients; one that is open to collaborating with other health and care professions and non-clinical researchers to improve our healthcare systems in the UK. Over the past 25 years, the paramedic role has rapidly expanded, embracing specialist, advanced and consultant roles across critical care, primary and urgent care, mental health, public health, strategic leadership, education and research, to name but a few areas. Paramedic practice has developed alongside a growth in our research capability. In fact, maybe we could go as far as to say that research has been a driver for this change by informing clinical decision making, challenging practice based on scant science, refining models of care and improving patient outcomes.

Over the past decade, the BPJ has become recognised as a place for sharing work ‒ an international platform for novice and experienced researchers and clinicians to contribute to the debates around evidence-based care and methodological innovation. The breadth, quality and diversity of submissions continue to increase year on year, reflecting the growth of our research-informed clinical community and the impact of our profession.

The next decade will undoubtedly bring further developments, whether they are technological, clinical, educational or organisational. But it will also bring opportunities to increase our evidence base, to challenge assumptions, to improve equity of care and to continue shaping a profession that is responsive, reflective and research-active.

The BPJ is moving with these changes by increasing the editorial board and adding to our Associate Editor team, working to ensure that we remain a journal that is current and responsive and that reflects the shape and needs of the paramedic profession and our collaborators.

As we celebrate these significant anniversaries in 2026, we want to take the time to extend our gratitude to our authors, reviewers, editorial board members, readers and the wider paramedic community. Your contributions have shaped not only this journal but the standing of the profession itself.

This year is a moment to recognise how far we have come and to look with confidence towards where we are going. The story of paramedicine is still being written. Who knows what challenges we will face within the NHS in the months and years to come, but the BPJ remains committed to demonstrating that our progress is grounded in robust evidence and inquiry to make the best choices we can with our patients and stakeholders.

With a strong foundation beneath us, we are well placed to embrace what comes next. So here’s to the next 25 years, to the next decade of the BPJ and to the first year of the Royal College of Paramedics.

**No AI has been used in the production of this editorial.**

